# Supplementary material for: Fatty-acid-induced FABP5/HIF-1 reprograms lipid metabolism and enhances the proliferation of liver cancer cells
Source: Commun Biol. 2020 Oct 30;3:638. doi: 10.1038/s42003-020-01367-5 (PMC7599230; doi:10.1038/s42003-020-01367-5)
Supplement: Supplementary file 1 — Supplementary Information [file 42003_2020_1367_MOESM1_ESM.pdf]

Supplementary Fig. 1

a

No.	Protein Reference	Score	Accession
**1**	Heat shock cognate 71 kDa protein isoform 2	200.22	24234686
**2**	Tubulin beta-2B chain	190.28	29788768
**3**	Aryl hydrocarbon receptor nuclear translocator isoform 3	180.27	30795242
**4**	Tubulin alpha-1B chain	150.27	57013276
**5**	Fatty acid-binding protein, epidermal	130.29	4557581
**6**	Ribosomal protein S3	110.19	15718687
**7**	60 kDa heat shock protein, mitochondrial	90.24	41399285
**8**	Tubulin beta-2C chain	80.23	5174735
**9**	Heat shock 70 kDa protein 1A/1B	80.21	194248072
**10**	Albumin preproprotein	80.17	4502027
**11**	Heat shock 70 kDa protein 1-like	70.25	124256496
**12**	Chaperonin containing TCP1, subunit 2	60.24	5453603
**13**	Elongation factor 2	60.23	4503483
**14**	Eukaryotic translation elongation factor 1 alpha 2	60.22	4503475
**15**	T-complex protein 1 subunit theta	60.22	48762932
**16**	78 kDa glucose-regulated protein	60.21	16507237
**17**	Elongation factor Tu, mitochondrial precursor	60.21	34147630
**18**	Actin, aortic smooth muscle	60.19	213688375

b

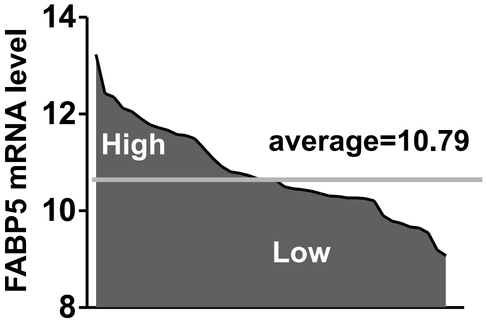

c

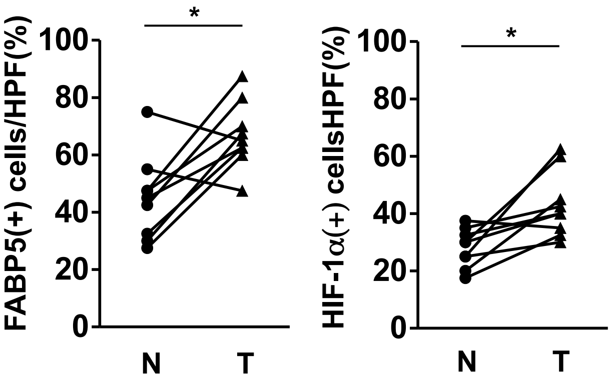

Supplementary Figure 1. Extended data for Fig.1
a, The lists of HIF-1α-interacting proteins. Filtering was performed based on score > 60. **b,** The GSE41804 dataset was divided into FABP5_High and _Low groups according to the average value. **c,** FABP5 and HIF-1α expressions in paired non-tumor/HCC tissues. The dots connected with line indicate tissues from the same patient.

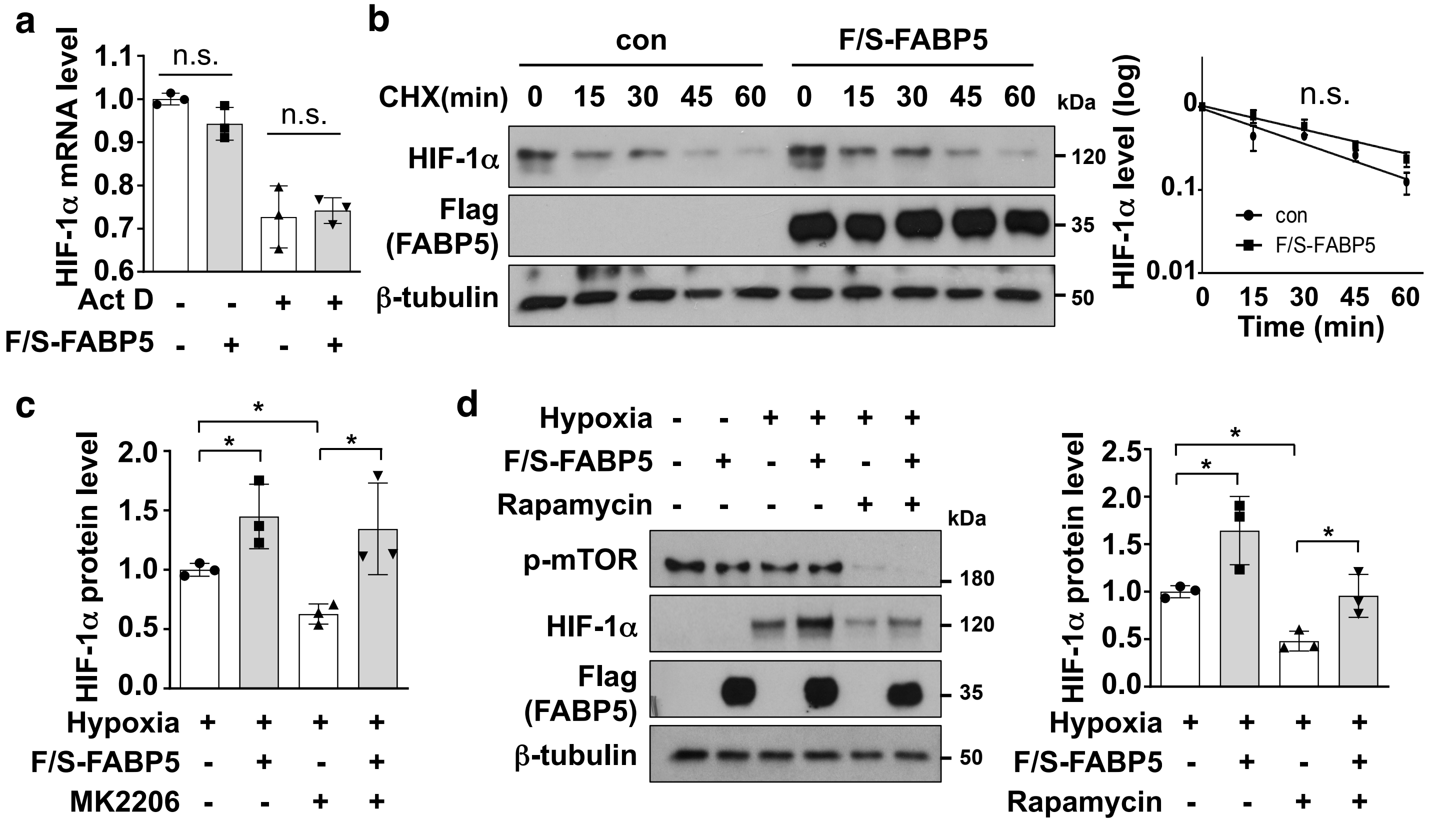

Supplementary Figure 2. Extended data Fig. 2

a, The transfected 293T cells were treated 100 μM Actinomycin D for 24 h. HIF-1α mRNA level was analyzed using RT-qPCR (mean ± SD, n = 3). **b,** The transfected 293T cells were incubated with DMOG 4h and then cycloheximide for the indicated time. Relative HIF-1α protein levels were plotted as the mean ± SD (n = 3), and the linear regression was plotted. **c,** The quantification data for Fig. 2f (mean ± SD, n = 3). **d,** The transfected 293T cells were incubated with 1 μM Rapamycin and hypoxia for 8 h. Cells lysates were immunoblotted and relative HIF-1α protein levels were plotted as the mean ± SD (n = 3).

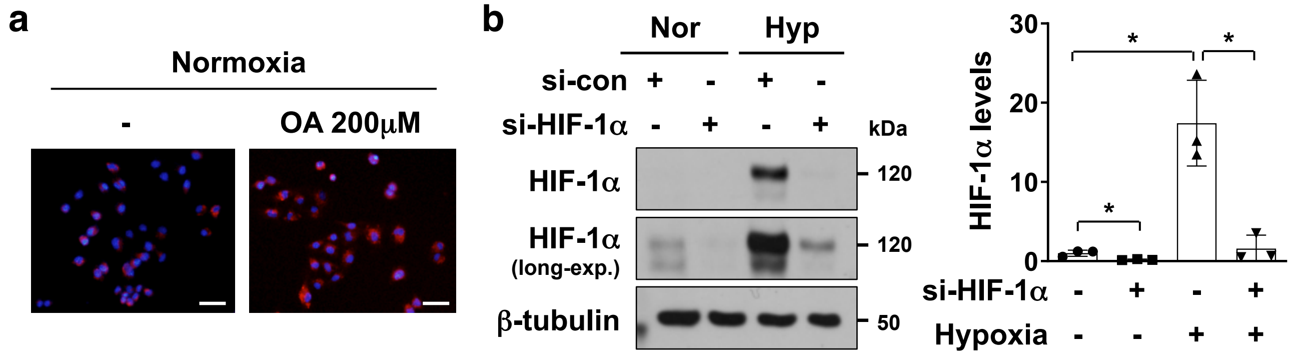

Supplementary Figure 3. Extended data for Fig. 5

a, Representative Nile red images. Cells were treated with OA, and stained with Nile red.

b, HepG2 cells were transfected with si-HIF-1α, and incubated under hypoxia for 8 h. Cells were subjected to immunoblotting and the blots were calculated by using Image J (mean ± SD, n = 3).

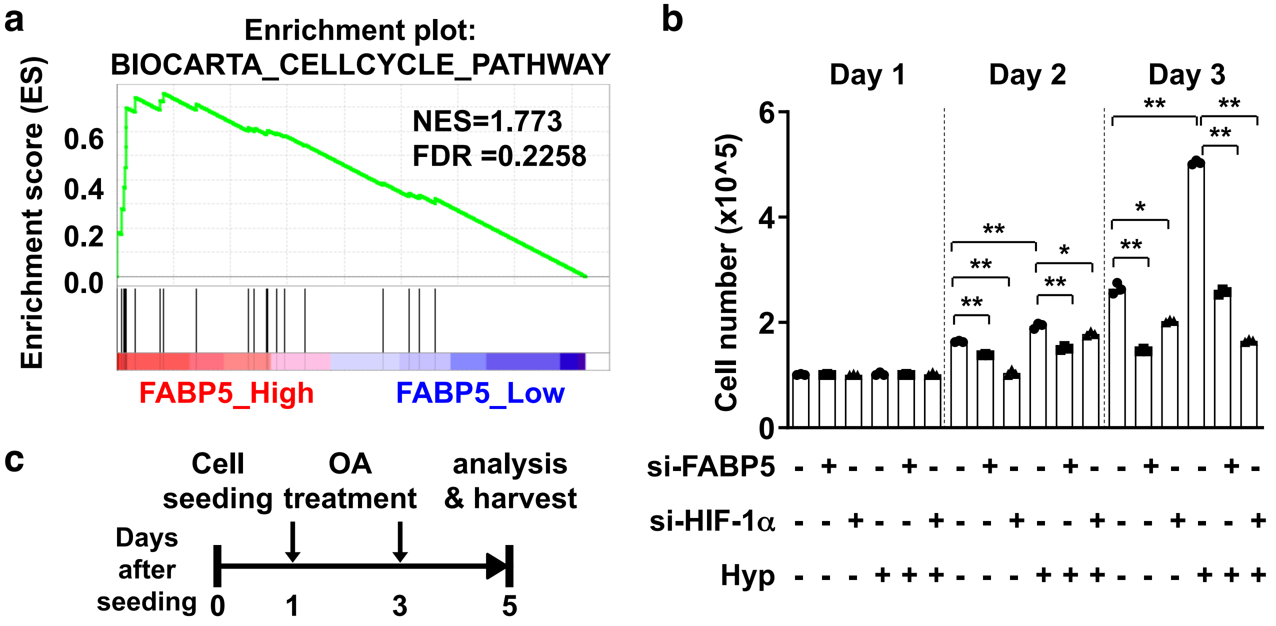

Supplementary Figure 4. Extended data for Fig. 6

a, GSEA enrichment plot for BIOCARTA_CELLCYCLE_PATHWAY gene set analyzed in HCC (GSE41804). **b,** The transfected HepG2 cells were incubated under hypoxic conditions for the indicated time. The total cell number was counted and presented as the means ± SD (n = 3). **c,** Scheme for 3D culture experiments was indicated.

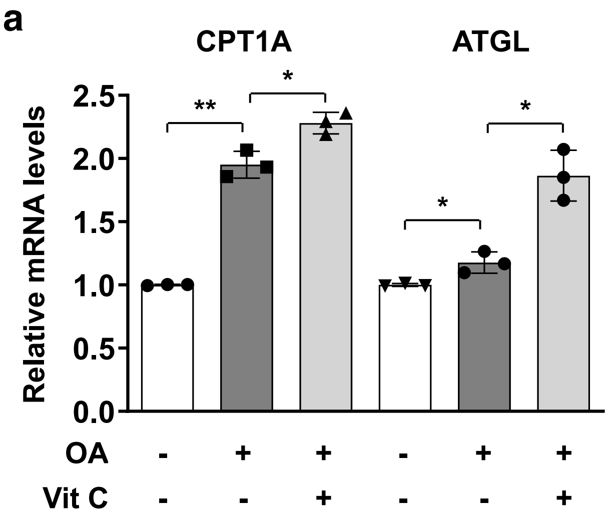

Supplementary Figure 5. Extended data for Fig. 7

a, Spheroids, which had been treated with OA and vitamin C, were subjected to RT-qPCR to measure *CPT1A* and *ATGL* mRNA levels. Data are presented as mean ± SD (n = 3).

Supplementary Fig. 6

Uncropped images of the western blots presented in the main and supplementary figures

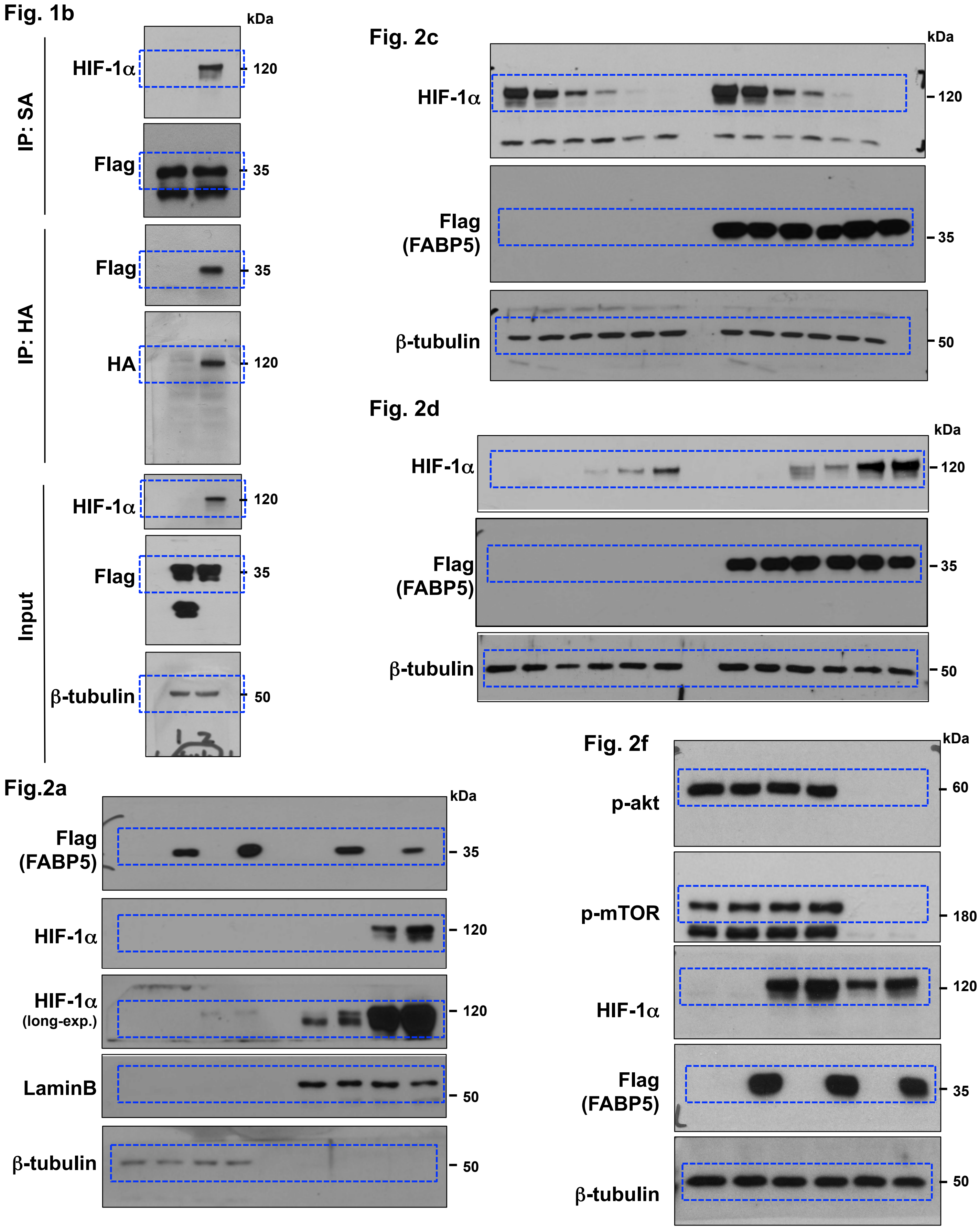

Supplementary Fig. 6

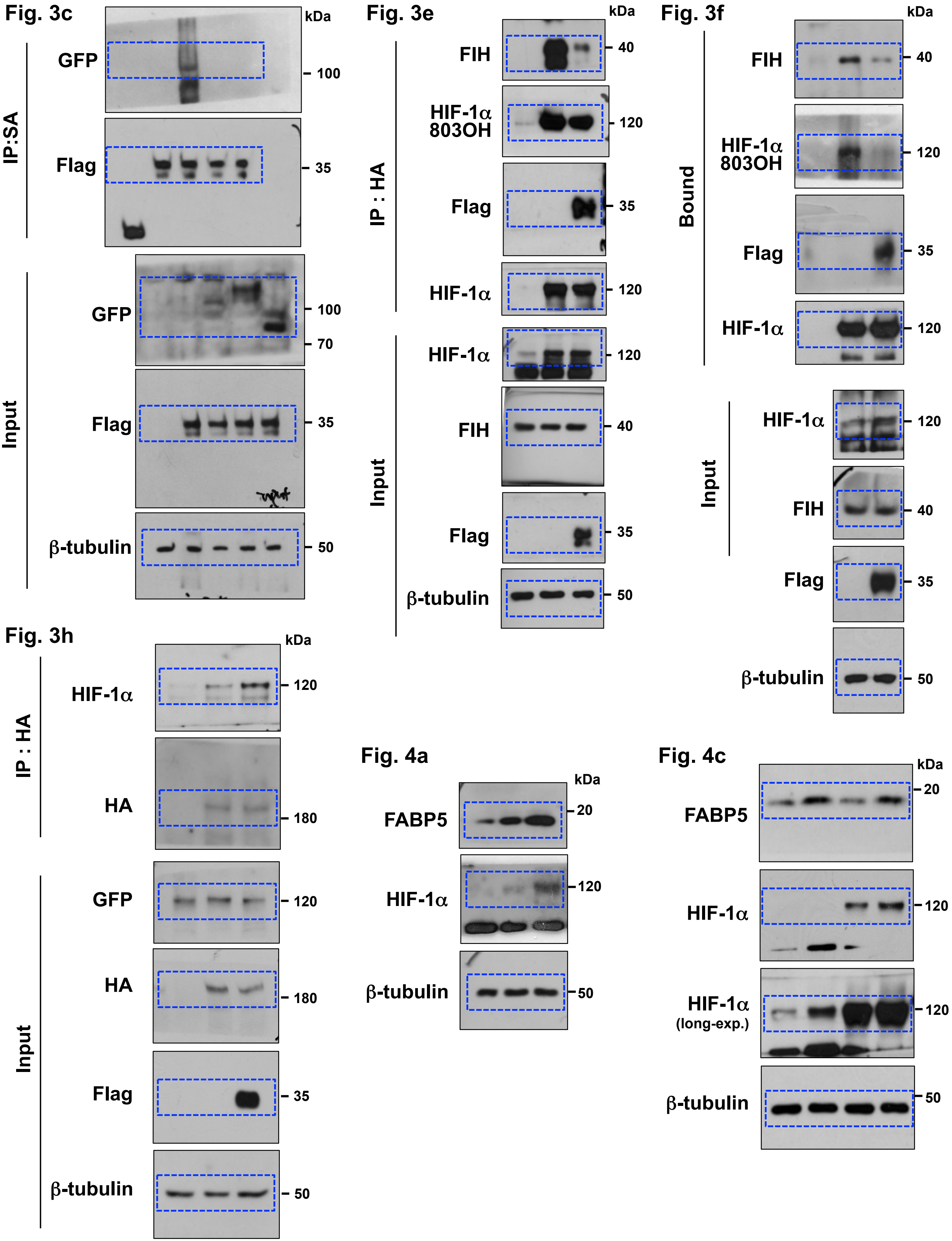

Supplementary Fig. 6

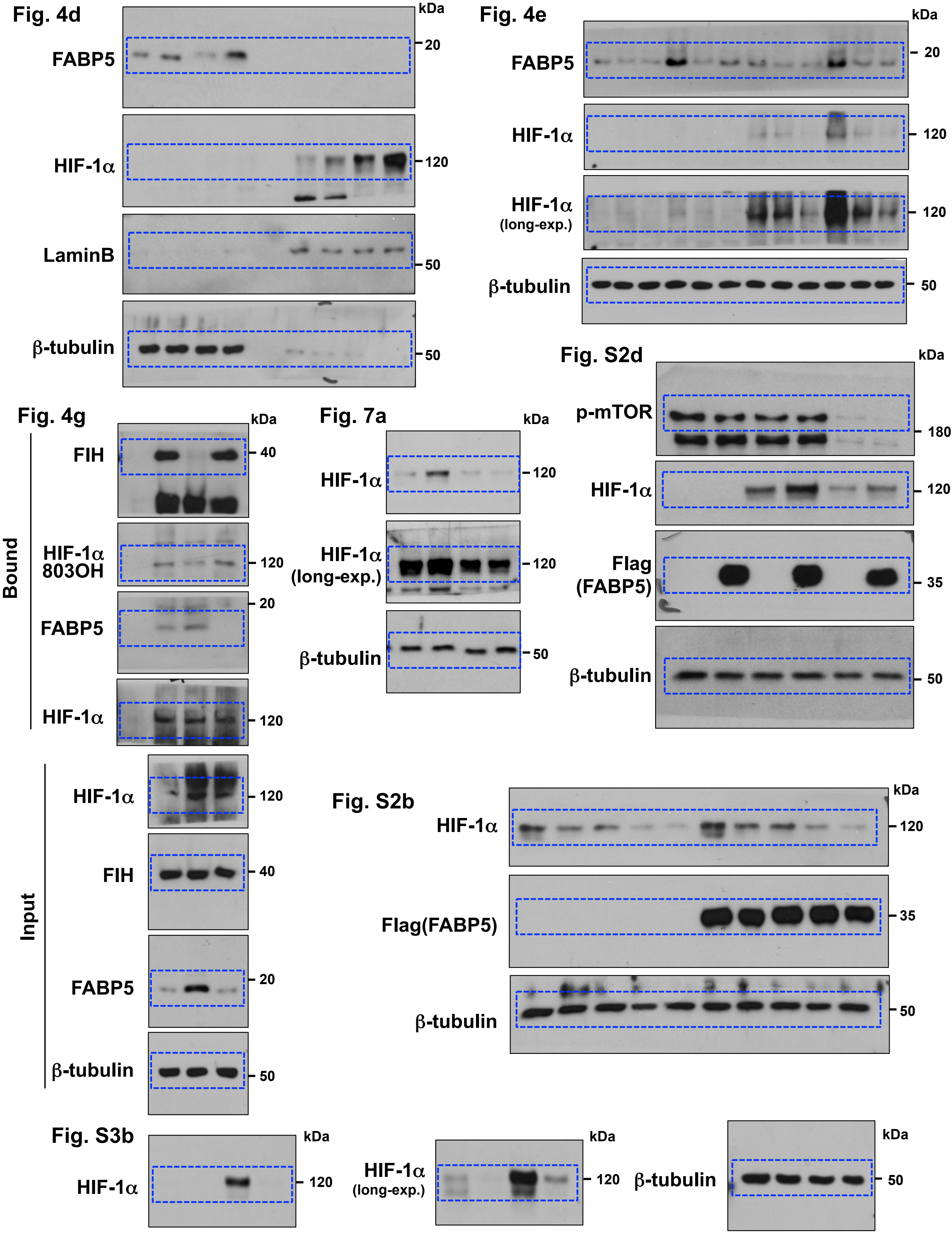

No.	Age	Sex	Organ	Diagnosis	pTNM	Stage
**1**	63	M	Liver	Hepatocellular carcinoma	T2N0M0	II
**2**	64	M	Liver	Hepatocellular carcinoma	T3N0M0	III
**3**	62	M	Liver	Hepatocellular carcinoma	T2N0M0	II
**4**	65	M	Liver	Hepatocellular carcinoma	T3N0M0	III
**5**	67	F	Liver	Hepatocellular carcinoma	T2N0M0	II
**6**	53	M	Liver	Hepatocellular carcinoma	T3N0M0	III
**7**	71	M	Liver	Hepatocellular carcinoma	T1N0M0	I
**8**	54	M	Liver	Hepatocellular carcinoma	T2N0M0	II
**9**	69	M	Liver	Hepatocellular carcinoma	T3N0M0	III
**10**	57	M	Liver	Hepatocellular carcinoma	T1N0M0	I
**11**	55	M	Liver	Hepatocellular carcinoma	T1N0M0	I
**12**	45	M	Liver	Hepatocellular carcinoma	T1N0M0	I
**13**	55	M	Liver	Hepatocellular carcinoma	T1N0M0	I
**14**	72	M	Liver	Hepatocellular carcinoma	T1N0M0	I
**15**	66	M	Liver	Hepatocellular carcinoma	T1N0M0	I
**16**	64	M	Liver	Hepatocellular carcinoma	T1N0M0	I
**17**	44	M	Liver	Hepatocellular carcinoma	T2N0M0	II
**18**	47	M	Liver	Hepatocellular carcinoma	T2N0M0	II
**19**	41	F	Liver	Hepatocellular carcinoma	T2N0M0	II
**20**	51	M	Liver	Hepatocellular carcinoma	T2N0M0	II
**21**	64	M	Liver	Hepatocellular carcinoma	T1N0M0	I
**22**	54	M	Liver	Hepatocellular carcinoma	T3bN0M0	IIIB
**23**	25	M	Liver	Hepatocellular carcinoma	T2N0M0	II
**24**	32	M	Liver	Hepatocellular carcinoma	T3N0M0	III
**25**	58	M	Liver	Hepatocellular carcinoma	T1N0M0	I
**26**	62	M	Liver	Hepatocellular carcinoma	T1N0M0	I
**27**	52	M	Liver	Hepatocellular carcinoma	T2N0M0	II
**28**	52	M	Liver	Hepatocellular carcinoma	T2N0M0	II
**29**	71	M	Liver	Hepatocellular carcinoma	T4N1M0	IVA
**30**	64	M	Liver	Hepatocellular carcinoma	T3bN0M0	IIIB
**31**	52	M	Liver	Hepatocellular carcinoma	T3bN0M0	IIIB
**32**	62	M	Liver	Hepatocellular carcinoma	T2N0M0	II
**33**	56	M	Liver	Hepatocellular carcinoma	T1N0M0	I
**34**	77	M	Liver	Hepatocellular carcinoma	T2N0M0	II
**35**	38	M	Liver	Hepatocellular carcinoma	T2N0M0	II
**36**	75	M	Liver	Hepatocellular carcinoma	T2N0M1	IVA
**37**	43	M	Liver	Hepatocellular carcinoma	T3aN0M0	IIIA
**38**	66	M	Liver	Hepatocellular carcinoma	T2N0M0	II
**39**	63	M	Liver	normal (adjacent to cancer, match of #1)		
**40**	64	M	Liver	normal (adjacent to cancer, match of #2)		
**41**	62	M	Liver	normal (adjacent to cancer, match of #3)		
**42**	65	M	Liver	normal (adjacent to cancer, match of #4)		
**43**	53	M	Liver	normal (adjacent to cancer, match of #6)		
**44**	71	M	Liver	normal (adjacent to cancer, match of #7)		
**45**	69	M	Liver	normal (adjacent to cancer, match of #9)		
**46**	55	M	Liver	normal (adjacent to cancer, match of #13)		
**47**	52	M	Liver	normal (adjacent to cancer, match of #28)		

Supplementary Table 1. Clinical information for utilized in IHC

No.	Age	Sex	Organ	Diagnosis
**1**	80	M	Liver	HBV-derived Hepatocellular carcinoma
**2**	57	M	Liver	HBV-derived Hepatocellular carcinoma
**3**	67	M	Liver	HBV-derived Hepatocellular carcinoma
**4**	57	M	Liver	HBV-derived Hepatocellular carcinoma
**5**	65	M	Liver	HBV-derived Hepatocellular carcinoma
**6**	40	M	Liver	HBV-derived Hepatocellular carcinoma
**7**	63	M	Liver	HBV-derived Hepatocellular carcinoma
**8**	77	M	Liver	HBV-derived Hepatocellular carcinoma
**9**	54	M	Liver	HBV-derived Hepatocellular carcinoma
**10**	60	M	Liver	HBV-derived Hepatocellular carcinoma
**11**	52	M	Liver	HBV-derived Hepatocellular carcinoma
**12**	53	M	Liver	HBV-derived Hepatocellular carcinoma
**13**	56	M	Liver	HBV-derived Hepatocellular carcinoma
**14**	71	M	Liver	HBV-derived Hepatocellular carcinoma
**15**	58	M	Liver	HBV-derived Hepatocellular carcinoma
**16**	72	M	Liver	HBV-derived Hepatocellular carcinoma
**17**	36	M	Liver	HBV-derived Hepatocellular carcinoma
**18**	61	M	Liver	HBV-derived Hepatocellular carcinoma
**19**	79	M	Liver	HBV-derived Hepatocellular carcinoma
**20**	53	M	Liver	HBV-derived Hepatocellular carcinoma

Supplementary Table 2. Clinical details used in analysis for mRNA levels (HBV : Hepatitis B virus)

	Forward 5'-3'	Reverse 3'-5'
Human si-FABP5 #1	GGAUCAUCCCUUUGG UUAAUAAATA	AUCCUAGUAGGGAAAC CAAUUAUUUAU
Human si-FABP5 #2	CAUUGUGAUGGUAAA AACCUCACC	UAGUGAACACUACCAU UUUUGGAGUGG
Human si-HIF-1a	GGGAUUAACUCAGUU UGAACUAACT	UACCCUAAUUGAGUCA AACUUGAUUGA

Supplementary Table 3. The si-RNA sequences for targeting FABP5 and HIF-1α

	Forward 5'-3'	Reverse 3'-5'
Human ACSL1-RT	GCTTTTGTGAAAGCAACAGAGA	GGCGAGAGGCAAGAAAGATA
Human ATGL-RT	GATCACATCCTGGAGCACCT	ACAGGCAGCATGTTGGAGA
Human BNIP3L-RT	TGCGAGGAAAATGAGCAGTC	TGCCATTGCTGCTGTTCATG
Human CA9-RT	TCCTGGGCTTCCAGCTCCCG	GCCCAGGAGGCAGGGTCAGT
Human CCND2-RT	GTGCTCCTCAATAGCCTG	TCTCTTTCGGCCCAACTG
Human CPT1A-RT	TGCTTTACAGGCGCAAACTG	TGGAATCGTGGATCCCAAA
Human DGAT2-RT	GGTCCTGTCCTTCCTTGT	AGTTGCCTGCCAGTGTAG
Human FABP5-RT	AGCAGCTGGAAGGAAGATGG	CTGATGCTGAACCAATGCAC
Human GPAT-RT	AACCCCAGTATCCCGTCTTT	CAGTCACATTGGTGGCAAAC
Human HIF-1a-RT	TGCAGAATGCTCAGAGAAAGCGAA	GCTGCATGATCGTCTGGCTGCT
Human LIPIN1-RT	TTTCCACGTCCGCTTTGGG	GTGGCCAGGTGCATAGGG
Human VEGF-RT	CGTGTACGTTGGTGCCCGCT	CCGCTCTGAGCAAGGCCCAC
Human ACSL1_ChIP-#1	CTCCCAACTATAGAGTCTGG	ACCTACCTCAGACAGGTGTC
Human ACSL1_ChIP-#2	TTTTGAGACAGAGTCTTGC	GGGAGGCTGAGGCAGAAGA
Human ACSL1_ChIP-#3	GACAGAAAAACCAGGGA	TCTCTAGCAGCTCAGTCCTT

Supplementary Table 4. The primer sequences for qPCR
