# Supplementary material for: Fatty-acid-induced FABP5/HIF-1 reprograms lipid metabolism and enhances the proliferation of liver cancer cells
Source: Commun Biol. 2020 Oct 30;3:638. doi: 10.1038/s42003-020-01367-5 (PMC7599230; doi:10.1038/s42003-020-01367-5)
Supplement: Supplementary file 3 — Description of Additional Supplementary Files [file 42003_2020_1367_MOESM3_ESM.pdf]

Description of Additional Supplementary Files

File Name: Supplementary Data 1
Description: All source data underlying the graphs presented in the main and supplementary figures.

File Name: Supplementary Data 2
Description: Raw data file for LC-MS/MS
