# Supplementary material for: Fatty-acid-induced FABP5/HIF-1 reprograms lipid metabolism and enhances the proliferation of liver cancer cells
Source: Commun Biol. 2020 Oct 30;3:638. doi: 10.1038/s42003-020-01367-5 (PMC7599230; doi:10.1038/s42003-020-01367-5)
Supplement: Supplementary file 6 — Reporting Summary [file 42003_2020_1367_MOESM6_ESM.pdf]

nature research

Corresponding author(s): Yang-Sook Chun

Last updated by author(s): Aug 26, 2020

Reporting Summary

Nature Research wishes to improve the reproducibility of the work that we publish. This form provides structure for consistency and transparency in reporting. For further information on Nature Research policies, see our Editorial Policies and the Editorial Policy Checklist.

Statistics

For all statistical analyses, confirm that the following items are present in the figure legend, table legend, main text, or Methods section.

n/a	Confirmed	
☐	☒	The exact sample size (n) for each experimental group/condition, given as a discrete number and unit of measurement
☐	☒	A statement on whether measurements were taken from distinct samples or whether the same sample was measured repeatedly
☐	☒	The statistical test(s) used AND whether they are one- or two-sided<br>*Only common tests should be described solely by name; describe more complex techniques in the Methods section.*
☐	☒	A description of all covariates tested
☒	☐	A description of any assumptions or corrections, such as tests of normality and adjustment for multiple comparisons
☐	☒	A full description of the statistical parameters including central tendency (e.g. means) or other basic estimates (e.g. regression coefficient) AND variation (e.g. standard deviation) or associated estimates of uncertainty (e.g. confidence intervals)
☐	☒	For null hypothesis testing, the test statistic (e.g. F, t, r) with confidence intervals, effect sizes, degrees of freedom and P value noted<br>*Give P values as exact values whenever suitable.*
☒	☐	For Bayesian analysis, information on the choice of priors and Markov chain Monte Carlo settings
☐	☒	For hierarchical and complex designs, identification of the appropriate level for tests and full reporting of outcomes
☐	☒	Estimates of effect sizes (e.g. Cohen's d, Pearson's r), indicating how they were calculated

Our web collection on statistics for biologists contains articles on many of the points above.

Software and code

Policy information about availability of computer code

Data collection	NCBI GEO database (GSE41804)
Data analysis	Gene Set Enrichment Analysis (http://broadinstitute.org/gsea)

For manuscripts utilizing custom algorithms or software that are central to the research but not yet described in published literature, software must be made available to editors and reviewers. We strongly encourage code deposition in a community repository (e.g. GitHub). See the Nature Research guidelines for submitting code & software for further information.

Data

Policy information about availability of data

All manuscripts must include a data availability statement. This statement should provide the following information, where applicable:

- Accession codes, unique identifiers, or web links for publicly available datasets
- A list of figures that have associated raw data
- A description of any restrictions on data availability

The article provides a full data generated or analyzed during this study.

Field-specific reporting

Life sciences study design

All studies must disclose on these points even when the disclosure is negative.

Sample size	No sample-size calculation was performed. Sample sizes were chosen by the pratical limitations of the protocol utilized.
Data exclusions	For analyzing HIF-1a-interacting proteins, we utilized anti-HA antibody to pull down the HA-HIF-1a N-terminal fragment (and HA plasmid as a control) and co-purified proteins were analyzed using liquid chromatography-tandem mass spectrometry. To identify proteins which are specifically interacting with HIF-1a, we excluded proteins pulled down in the control group. Filtering was performed based on score > 60 for each group.
Replication	All experiments were reproduced to support reliable conclusions stated in the manuscript. For each experiment, biologically independent samples were plated in triplicate and showing consistency in all multiple batches.
Randomization	No specific method of randomization was used in the study.
Blinding	Blinding is not applicable to the described experimental designs.

Reporting for specific materials, systems and methods

We require information from authors about some types of materials, experimental systems and methods used in many studies. Here, indicate whether each material, system or method listed is relevant to your study. If you are not sure if a list item applies to your research, read the appropriate section before selecting a response.

Materials & experimental systems

n/a	Involved in the study	
☐	☒	Antibodies
☐	☒	Eukaryotic cell lines
☒	☐	Palaeontology and archaeology
☒	☐	Animals and other organisms
☐	☒	Human research participants
☒	☐	Clinical data
☐	☒	Dual use research of concern

Methods

n/a	Involved in the study	
☒	☐	ChIP-seq
☒	☐	Flow cytometry
☒	☐	MRI-based neuroimaging

Antibodies

Antibodies used	anti-FABP5 (R&D systems); anti-p-akt, anti-p-mTOR, and anti-Ki-67 (Cell Signaling); anti-flag (Sigma-Aldrich); anti-B-tubulin and anti-Lamin B (Santa Cruz Biotechnology); anti-GFP (Thermor Fisher Scientific); anti-HA (GeneTex). Anti-HIF-1a was generated against human HIF-1a in rabbits and a monoclonal anti-hydroxylated Asn 803 of HIF-1a was raised in mouse as described previously.
Validation	All manufactured antibodies were validated by manufacturers. Generated anti-HIF-1a and anti-hydroxylated Asn 803 of HIF-1a were validated and used in previous studies (Chun YS, Choi E, Kim GT, Lee MJ, Lee SE, et al. Zinc induces the accumulation of hypoxia-inducible factor (HIF)-1alpha, but inhibits the nuclear translocation of HIF-1 beta, causing HIF-1 inactivation. Biochem Biophys Res Commun 2000;268:652-6., Li SH, Shin DH, Chun YS, Lee MK, Kim MS, Park JW, A novel mode of action of YC-1 in HIF inhibition: stimulation of FIH-dependent p300 dissociation from HIF-1 alpha, Mol Cancer Ther, 2008 Dec; 7(12):3729-38).

Eukaryotic cell lines

Policy information about cell lines

Cell line source(s)	HepG2 (a human hepatocellular carcinoma cell, Korea Cell Bank) and HEK293 (a human embryonic kidney cell, American Type Culture Collection)
Authentication	The cell lines were authenticated by manufacturers.
Mycoplasma contamination	All cell lines were prevented from mycoplasma contamination by using mycoplasma elimination reagent regularly (Myco-Guard PLUS, Biomax).
Commonly misidentified lines (See ICLAC register)	No commonly misidentified cell lines were used in the study.

Human research participants

Policy information about studies involving human research participants

Population characteristics	Population characteristics (age, gender, organ, diagnosis) were described in suppplementary table 1 and 2.
Recruitment	Human hepatocellular carcinoma tissues were obtained with consent under approval by the Institutional Review Board (IRB) committees of the Seoul National University Hospital (SNUH).
Ethics oversight	IRB committees of the SNUH.

Note that full information on the approval of the study protocol must also be provided in the manuscript.

Dual use research of concern

Policy information about dual use research of concern

Hazards

Could the accidental, deliberate or reckless misuse of agents or technologies generated in the work, or the application of information presented in the manuscript, pose a threat to:

No	Yes	
☒	☐	Public health
☒	☐	National security
☒	☐	Crops and/or livestock
☒	☐	Ecosystems
☒	☐	Any other significant area

Experiments of concern

Does the work involve any of these experiments of concern:

No	Yes	
☒	☐	Demonstrate how to render a vaccine ineffective
☒	☐	Confer resistance to therapeutically useful antibiotics or antiviral agents
☒	☐	Enhance the virulence of a pathogen or render a nonpathogen virulent
☒	☐	Increase transmissibility of a pathogen
☒	☐	Alter the host range of a pathogen
☒	☐	Enable evasion of diagnostic/detection modalities
☒	☐	Enable the weaponization of a biological agent or toxin
☒	☐	Any other potentially harmful combination of experiments and agents
